# Impact of the donor-recipient gender matching on the graft survival from live donors

**DOI:** 10.1186/s12882-019-1670-x

**Published:** 2020-01-06

**Authors:** Gholamhossein Naderi, Amin Azadfar, Seyed Reza Yahyazadeh, Fatemeh Khatami, Seyed Mohammad Kazem Aghamir

**Affiliations:** 1 Shariati Hospital, Department of Urology, Tehran University of Medical Sciences, Tehran, Iran; 2 Urology Research Center, Tehran University of Medical Sciences, Tehran, Iran

**Keywords:** Kidney transplant, Gender match, Survival, Dialysis

## Abstract

**Background:**

Several factors such as recipient age, BMI, serum cratinine, and positive history of dialysis are important in predicting graft survival among kidney transplant recipients. One factor affecting the transplant outcomes is donors and recipients gender, which is usually ignored.

**Methods:**

A total of 1113 kidney transplant recipients were studied in this retrospective cohort study. Several factors were taken into account for graft survival and outcomes such as donors and recipients gender and age in addition to common recipient factors like cratinine, eGFR, BMI, and positive history of dialysis.

**Results:**

The most successful transplant based on donor-recipient gender was observed in male donor to male recipient, and then male donor to female recipient. In female transplant recipients, level of serum cratinine and eGFR, positive dialysis history before transplant, and low donor hemoglobin level can be considered as good prognostic factors recommended for kidney transplant survival.

**Conclusions:**

Our results suggested gender matching for kidney transplant. Only in some exceptional conditions, male donor to female recipient kidney transplant may be successful and female donors to male recipients are not suggested, especially in aged patients with the history of dialysis.

## Introduction

For most patients with end-stage renal disease (ESRD), the only treatment strategy is renal transplantation. Kidney transplantation has become a better cost-effective alternative to dialysis as a result of several improvements in early graft survival and long-term graft function. The first kidney transplantation was performed about half a century ago in which the transplant was performed from a live donor to his identical twin. After that, the concept of living and non-living unrelated donor resulted in increasing the number of organ donation in ESRD, and the number of kidney transplantations has escalated over the last ten years. More than two thousand kidney transplants have been reported in Iran, among which 50% were deceased (brain death) cases.

Several factors may have an impact on patients and/or graft survival, and also on transplant outcomes. Donor factors such as age, female gender, brain death of cerebrovascular cause, and prolonged criteria donor status had a noteworthy effect on the renal graft function [1–3]. Both recipient and donor Gender is one of the candidate elements that can better determine the graft outcomes, and also the gender match is suggested. Similar graft survival rates for males and females have been reported and a systematic review on gender differences in kidney transplantation identified 14 conducted studies with contradicting results [4, 5]. However, more recently, a study from the Collaborative Transplant Study confirmed that female recipients of male donor kidneys had the worst graft survival by passing the first year and up to ten years post-transplant [6].

In this study, we aimed to investigate the impact of gender match between kidney donor and recipients. Moreover, we had the purpose of evaluating the predictive markers of graft survival.

## Patients and methods

In this retrospective cohort study, among 2000 graft recipient patients who were registered in transplant center of Shariati Hospital transplant data bank from 2002 to 2018, 1113 renal graft recipients were chosen for our research. All selected patients were similar in being living donors; however, they were different in having the same donor gender and relative or non-relative donor. Patients with positive cross-match, incompatible blood group, age < 18 years old, multiple organ transplantation history, positive chronic viral B, C hepatitis, HIV, pregnancy, and diabetes were excluded from the study. In addition, patients with positive history of liver diseases such as Cirrhosis, Autoimmune hepatitis (including Gamma globulin serum & FANA), primary biliary cirrhosis, biliary obstruction, hemochromatosis, alpha-1- antitrypsin deficiency, Wilson’s disease (including serum ceruloplasmin, transferrin saturation percentage), Chronic disabling diseases (severe cardiac dysfunction, Chronic obstructive pulmonary disease, malignancy), and known cancers were excluded from the study.

Delayed graft function was defined by reduction in urine volume (≤400 ml/24 h) or requiring dialysis one week after graft receipt. The biopsy was done for patients who were at the risk of rejection with more than 30% increase in their basic levels of cratinine. Anti-rejection medication was Methylprednisolone and on the occasion of its resistance, Anti-thymocyte globulin (ATG) was the alternative. Transplant rejection was defined as dialysis need for more than 30 days, death, or graft excision. Patients included in this study were categorized into four distinct groups as group 1 (donor: male, recipient: male), group 2 (donor: male, recipient: female), group 3 (donor: female, recipient: male), and finally group 4 (donor: female, recipient: female). Clinical data such as duration of dialysis before kidney transplantation, serum cratinine, and eGFR (Estimated Glomerular Filtration Rate) for transplant recipients were considered. In addition, some demographic information belonged to both recipients and donors like age, BMI in both donor, and recipient and donor’s hemoglobin were considered. Graft rejection, needing dialysis again, and death were checked for several factors using multiple logistic regression models.

### Statistical analysis

Actuarial survival was assessed by Kaplan-Meier test, and the log derivation of the survival percentage was employed for the half-life predicting of grafts and/or patients. Differences in survival were done through log-rank test, and *p*-value less than 0.05 was considered as significant. To determine the factors having an independent impact on graft survival, a Cox proportional hazard analysis was utilized.

## Results

The average creatinine levels (mean and standard deviation (SD)) were 1.65 ± 1.46 mg/dl, 1.63 ± 1.29 mg/dl, 1.37 ± 0.69 mg/dl and 1.37 ± 0.66 mg/dl for one week, one month, six months, and one year post-transplant, respectively. The mean eGFR was 58.2 ± 22.2 cm^3^/min in the first month, 62.0 ± 21.4 cm^3^/min after six months, and 62.15 ± 21.1 cm^3^/min by passing one years from transplant. These decreasing trends in the amount of serum creatinine and the increasing trend in serum eGFR were meaningful (*p*-value = 0.039). Table 1 shoes the characteristics of both graft donors – recipients, and Table 2 indicates the numbers of patients in each gender match groups. Additional data on serum creatinine and eGFR levels of transplant recipients and donors hemoglobin are shown in Table 3 over several times of sampling before and after the transplantation.

**Table 1:** Characteristics considered for both graft donors and recipients

	Kidney transplant Donor	Kidney transplant Recipient
Mean age (year)	28.20 ± 5.34	35.02 ± 15.63
Female (percent)	202 (18.2%)	438 (39.4%)
BMI	24.02 ± 4.40	23.29 ± 5.41
Dialysis Before Graft	–	852 (76.6%)
Arterial anastomosis	–	972 (87.4%)
Venous anastomosis	–	1101 (99.2%)
DGF	–	103 (9.3%)

**Table 2:** The numbers of patients in each gender match groups

Donor-Recipient Gender	Number	Percent
Male to Male	1123	53/1%
Male to Female	557	26.9%
Female to Female	179	8.8%
Female to Male	213	9.5%
Total	2072	100%

**Table 3:** Fluctuation of recipients’ serum creatinine and eGFR vs. donors’ hemoglobin

	Time	Male to Male (Means ± SD)	Male to Female (Means ± SD)	Female to Female (Means ± SD)	Female to Male (Means ± SD)	Total	*p*-value
Serum Creatinine in Graft Recipients	One week after transplant	1.72 ± 1.48	1.60 ± 1.46	1.59 ± 1.66	1.65 ± 1.56	1.67 ± 1.66	0.474
	One month after transplant	1.72 ± 1.41	1.51 ± 1.23	1.53 ± 1.40	1.56 ± 0.89	1.63 ± 1.32	0.009*
	Six months after transplant	1.42 ± 0.65	1.24 ± 0.69	1.21 ± 0.45	1.61 ± 0.93	1.37 ± 0.69	0.000*
	One year after transplant	1.42 ± 0.58	1.22 ± 0.56	1.32 ± 0.91	1.57 ± 0.92	1.37 ± 0.66	0.000*
eGFR in Graft Recipients	One month after transplant	61.64 ± 21.76	53.16 ± 21.58	52.69 ± 22.04	58.78 ± 21.73	58.31 ± 22.08	0.000*
	Six months after transplant	66.35 ± 21.28	57.10 ± 19.68	55.23 ± 19.35	57.69 ± 23.07	62.04 ± 21.39	0.000*
	One year after transplant	66.12 ± 19.99	57.59 ± 20.39	55.44 ± 22.65	58.85 ± 22.60	62.18 ± 21.05	0.000*
Hemoglobin of Graft Donors	Before surgery	15.10 ± 1.28	15.05 ± 1.30	12.89 ± 1.24	12.83 ± 1.17	14.66 ± 1.54	0.000*
	After surgery	14.00 ± 1.33	13.92 ± 1.42	12.10 ± 1.30	12.03 ± 1.26	13.61 ± 1.54	0.000*
	Two days after surgery	13.86 ± 1.49	13.82 ± 1.34	11.83 ± 1.32	11.71 ± 1.21	13.45 ± 1.63	0.000*

*p-value< 0.05 is considered as significant. *P*-value is estimated by Anova

Side effects and complications after surgery were just seen in 4.6% of the patients. Graft rejection was reported in about 51 cases (4.6%) of graft recipients, and 130 (11.7%) of the patients were made to have dialysis after transplantation. There were 41 (3.7%) post-transplant death cases. Surgery Consequences, side effects, graft rejection, dialysis return, and death caused by transplant in recipients are presented in Table 4, based on donor-recipient gender.

**Table 4:** Consequences of surgery like side effects, graft rejection, dialysis return, and death in transplant recipients

Consequence	Frequency	*p*-value
Side Effects		
Male to Male	2.3%	0.068
Male to Female	4.0%	
Female to Male	3.8%	
Female to Female	4.5%	
Graft Rejection		
Male to Male	4.0%	0.087
Male to Female	6.4%	
Female to Male	3.8%	
Female to Female	4.0%	
Dialysis Return		
Male to Male	10.5%	0.009*
Male to Female	15.0%	
Female to Male	8.4%	
Female to Female	12.1%	
Death		
Male to Male	3.4%	0.517
Male to Female	4.3%	
Female to Male	3.6%	
Female to Female	3.2%	

*p-value< 0.05 is considered as significant. Chi 2 test is used for earning *p*-value

**Fig. 1:**
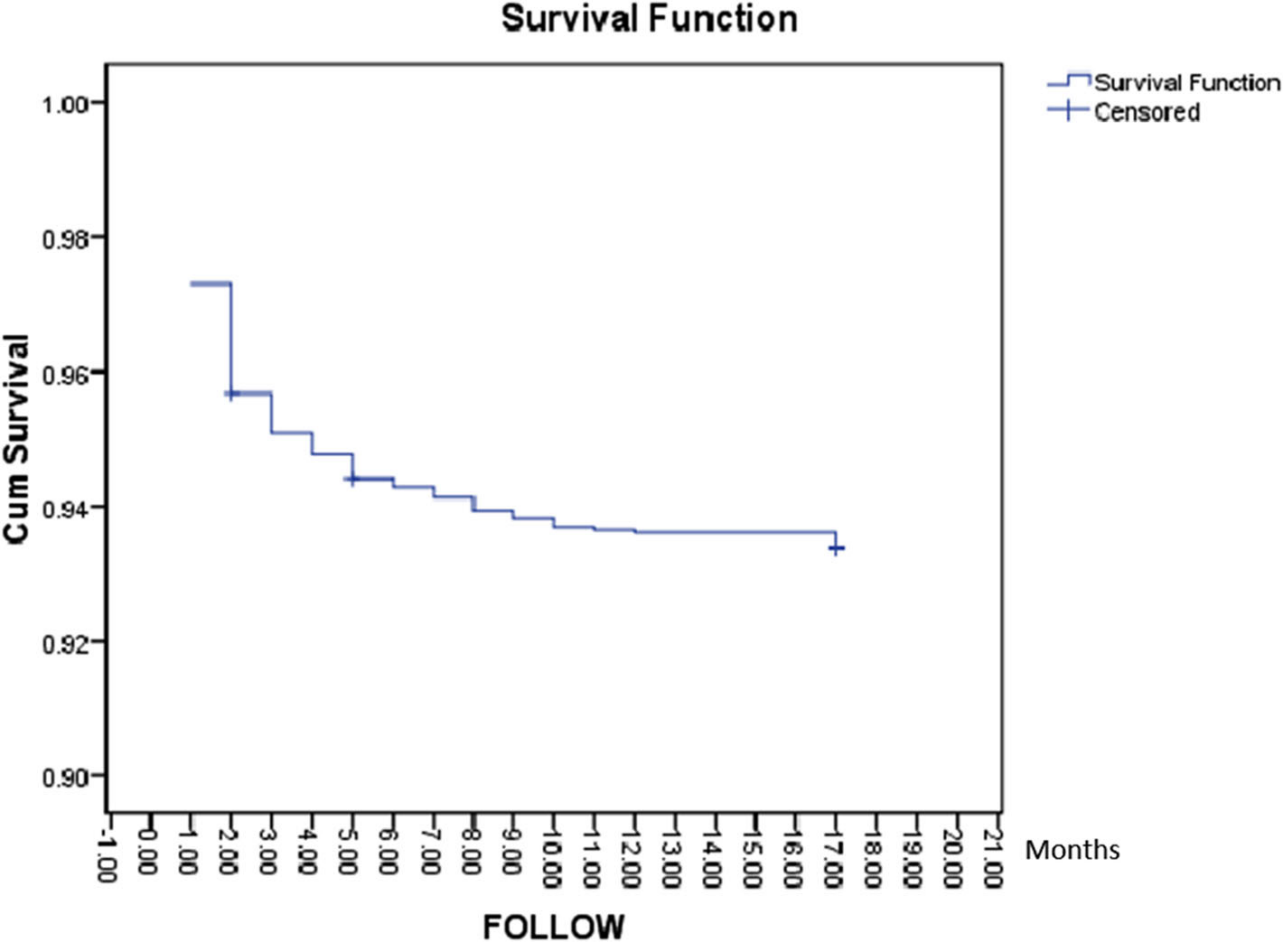
Survival rate of kidney transplant recipients

Based on the results of Kaplan-Meier test, the survival percentage was 97.3, 95.7, 95.1, 94.8, 94.4, and 93.7% one year, two, three, four, five, and ten years’ post-transplant, respectively (Fig. 1). The patient’s distribution in our four defined groups based on donor-recipient gender was 606 (54.4%) in group 1, 304 (27.4%) in group 2, 105 (99.4%) in group 3, and 98 (8.8%) in group 4.

Predictive factors for mortality, graft rejection, and back to dialysis in both donor and recipient genders were age (esp. in transplant recipients), serum creatinin, eGFR, level of donor hemoglobuline, history, and duration of dialysis before kidney transplant. The result of multiple logistic regression analysis of graft rejection, dialysis return, and death recipients are presented in Table 5. The multiple logistic regression model for graft rejection indicated female recipient (*p*-value: 0.035), delayed graft function (DGF) (p-value: 0.001), recipients age (p-value: 0.050), primary serum level of creatinine and eGFR (p-value: 0.001), and donor hemoglobin levels (*p* value: 0.043) are critical elements of graft rejection. Based on the results shown in Table 5, we found that in the similar conditions for CFR recipient sex and recipient age, if matched all condition, females would have a 1% chance of graft rejection for every 1 unit increase in hemoglobin.

**Table 5:** Multiple logistic regression models for several factors and graft rejection requiring dialysis return and death

	Candidate Variable	Beta coefficient	*p*-value	Probability Ratio
Graft Rejection	Recipient’s Gender	−0.437	0.040	0.646
	Age of Recipient	0.019	0.010*	1.019
	Creatinine/eGFR	−0.341	0.001*	0.711
	Donor’s hemoglobin level	0.019	0.001*	1.019
Dialysis Return	Recipient’s Gender	−0.602	0.002*	0.548
	Age of Recipient	0.038	0.001*	1.039
	Creatinine/eGFR	−0.275	0.001*	0.760
	Donor’s hemoglobin level	0.148	0.016*	1.159
	Arterial Anastomosis	0.171	0.650	1.187
	DGF	0.564	0.146	1.758
	Donor’s Age	−0.022	0.177	0.978
Death	History of Dialysis	0.417	0.453	1.518
	Age of recipient	−0.042	0.010*	1.959
	Creatinine/eGFR	−0.305	0.001*	0.749
	BMI of Recipient	0.009	0.009*	0.959
	Creatinine/eGFR	−0.067	0.642	0.935

**Fig. 2:**
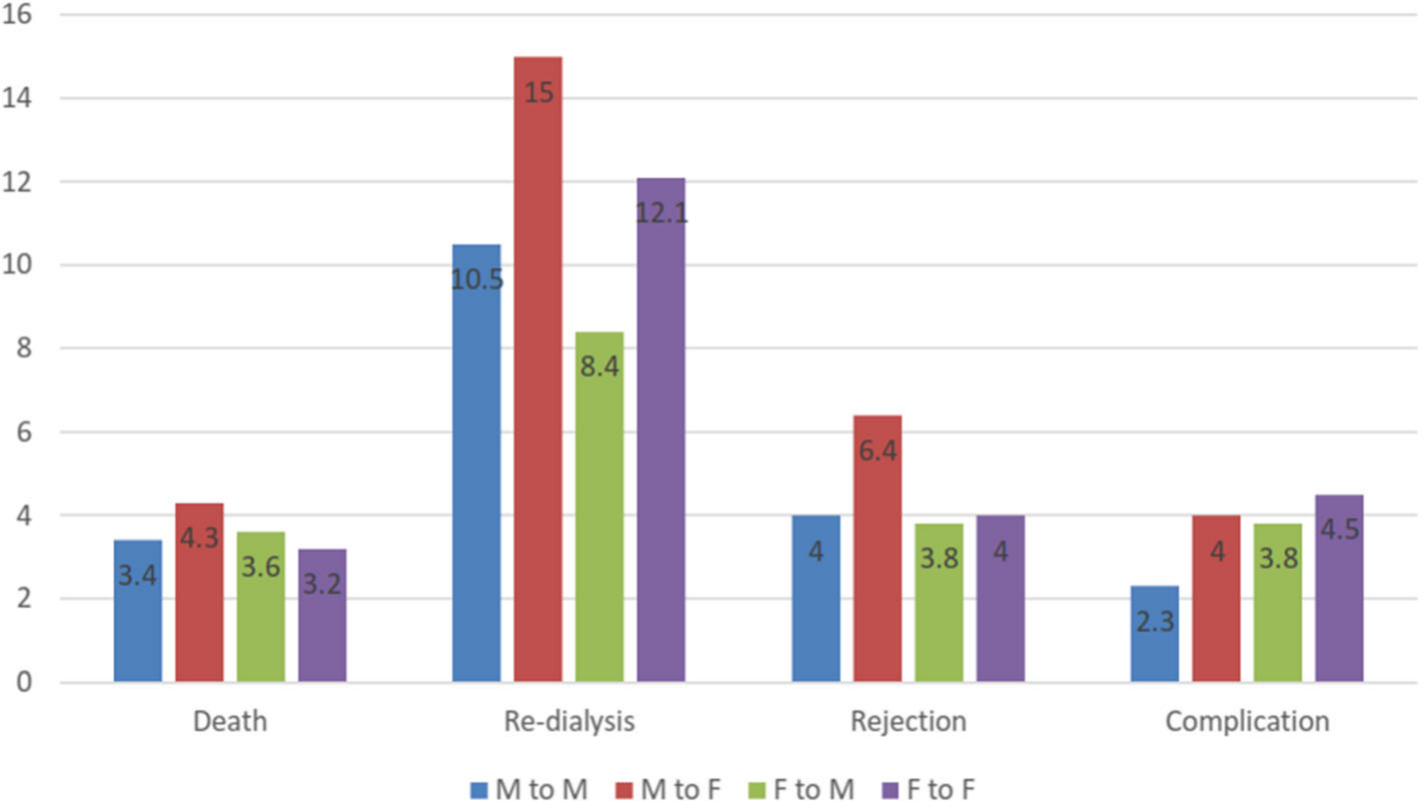
Frequency of death, back to dialysis, graft rejection and other complications based on gender math

The multiple regression models showed that kidney transplant recipients with the history of dialysis, older age, higher Creatinine/eGFR, and higher hemoglobin levels in their donors are more susceptible to the return of dialysis after transplantation. In addition, higher Creatinine/eGFR, BMI and older age of recipients (≥35 years) can decrease the risk of transplant survival, and also increase the risk of death in graft recipients. Figure 2 represents the frequency of death due to dialysis, graft rejection, and other complications based on the gender math.

## Discussion

Our observational study indicated that gender match between kidney donor and recipient is an important graft survival determinant. More than sex match, transplant survival was dependent on younger age of recipient and having the history of dialysis before transplant. Several factors such as the incidence of post-transplant hyperglycemia, its cardiovascular disease as the Pre-transplant characteristics, and particularly, the glycemia during the first month post-transplant identified patients with the risk of post-transplant diabetes, suggested by some studies can change the long-term survival in renal transplant recipients with graft function [7]. For example, the incidence of post-transplant hyperglycemia due to pre-transplant cardiovascular disease, and particularly, glycemia during the first month post-transplant identified patients at risk of post-transplant diabetes. Our result showed that the age of recipient < 35 is more critical than the age of donor. For cardiac and hepatic grafts, no significant effect of donor gender on the proportion of patients treated for rejection episodes was seen, and adverse effects of female donor gender on different organs is much less uniformed [8]. Donor’s age can be the potential confounder because a gender effect on graft survival was also observed for cardiac allograft. The age of donors> 60 years old or other algorithms were considered to simplify the identification of organs with elevated risk of transplant failure [9]. Our findings showed that the age of recipients< 35 is more critical than the age of donors. Age matching can possibly increase the positive results of transplantation, particularly when kidneys from older donors are used [10]. By considering the age and gender as principles for the optimal donor/recipient selection may be taken in organ allocation [11, 12]. Some structural and functional changes occurring in kidney with age increase can change the efficacy of transplantation.

Peter Stenvinkel et al. suggested that as inflamed female’s patients have a better outcome compared to inflamed males, because sex hormones may have important cardioprotective effects that limit the effect of inflammation on vascular injury in female patients with end-stage renal disease (ESRD). This can be the reason that female recipients, even from male donors, showed higher transplant outcomes in comparison to male recipients (from female donors). Additional studies must conclude whether sex- and age-specific immunosuppressant is warranted for kidney graft recipients [13–15]. Despite the conflicting data regarding the influence of gender on chronic kidney disease, it was shown by Idan Goldberg and Ilan Krause that the prevalence of chronic kidney disease tends to be higher in women, whereas the disease is more severe in men [16]. In the pre-lung allocation score era, female gender was not connected with better survival. Female recipients showed considerably improved survival rate over five years compared to males [17]. However, there are some suggestions that the impressive long-term graft survival benefit of male donor-female recipient versus female donor-male recipient, and of male donor-female recipient versus matched groups (male-male, female-female) in transplant, can be resulted from some donor quality and recipient characteristics as the confounding elements [18–20].

Our data indicated that women are good donors on the basis of hemoglobin; however, on the basis of contradictory gender, we conclude that men are better donors. In female donors, individuals with lower hemoglobin are considered to be more appropriate as the donor than women with higher hemoglobin (within group), and in men with lower hemoglobin compared to higher hemoglobin. Time of being in waiting list of transplantation and duration of dialysis can be confounders such as the donors and recipients age. Our data suggested that in addition to recipients’ age and gender matching as well as positive history of dialysis can be a prognostic element of transplant survival. Several previous studies pointed out the natural history of permanent renal dysfunction and severe liver failure in liver transplant recipients that can be helpful in the progress of non-nephrotoxic immunosuppressive regimens for high-risk liver transplant recipients [21, 22].

A paired donor kidney analysis indicated that waiting time on dialysis can be the strongest modifiable risk factor for renal transplant outcomes **[**23**]**. The advantage of living-donor versus cadaveric-donor transplantation can be the reason for waiting time. Cadaveric renal transplant recipient with an end-stage renal disease time ≤ 6 months has the equivalent graft survival of living donor transplant recipients compared to those waiting on dialysis waiting list for ≥2 years **[**24**]**. In fact, increased time on dialysis before kidney transplantation is linked with the reduction in survival of transplant recipients.

## Conclusion

By far, the most successful transplants, based on donor-recipient gender, were seen in male donors to male recipients, and then male donors to female recipients. Contradictory, the most unsuccessful transplant was observed when the donor was female and the recipient was male. In female transplant recipients, the level of serum cratinine, and eGFR, positive dialysis history before transplant, and low donor hemoglobin levels can be good prognostic factors in kidney transplant survival. By Judging these results based on hemoglobin yields, if we take Gender into account, we get inconsistent results. Therefore, further studies are needed to complete this section.”

## Data Availability

Information, data, and photos will be provided upon request by Dr. Seyed Mohammad Kazem Aghamir (mkaghamir@tums.ac.ir).

## Abbreviations

ATG: Anti-thymocyte globulin
BMI: *Body mass index*
CKD: Chronic *kidney* disease
CRF: Chronic *renal* failure
eGFR: Estimated Glomerular Filtration Rate
ESRD: End-stage renal disease
LDKT: Living donor *kidney transplantation*

## References

[1] Auglienė R, Dalinkevičienė E, Kuzminskis V, Jievaltas M, Peleckaitė L, Gryguc A (2017). Factors influencing renal graft survival: 7-year experience of a single center. Med..

[2] Muhammad AS, Naicker S (2018). HLA matching and kidney allograft function, experience from a south African transplant Centre. Trop J Nephrol.

[3] Locke JE, Durand C, Reed RD, MacLennan P, Mehta S, Massie A (2016). Long-term outcomes after liver transplantation among human immunodeficiency virus infected recipients. Transplant..

[4] Horvat LD, Shariff SZ, Garg AX, Network DNOR (2009). Global trends in the rates of living kidney donation. Kidney Int.

[5] Jindal RM, Ryan JJ, Sajjad I, Murthy MH, Baines LS (2005). Kidney transplantation and gender disparity. Am J Nephrol.

[6] Gratwohl A, Döhler B, Stern M, Opelz G (2008). HY as a minor histocompatibility antigen in kidney transplantation: a retrospective cohort study. Lancet.

[7] Hariharan S (2001). Long-term kidney transplant survival. Am J Kidney Dis.

[8] Zeier M, Döhler B, Opelz G, Ritz E (2002). The effect of donor gender on graft survival. J Am Soc Nephrol.

[9] Alfrey E, Lu A, Carter J, Dafoe D, editors. Matching does not improve outcome from aged marginal kidney donors. Transplant Proc; 2001. 10.1016/s0041-1345(00)02443-x 11267238

[10] Kwon O-J, Kwak J-Y, Kang C-M, editors. The impact of gender and age matching for long-term graft survival in living donor renal transplantation. Transplant Proc; 2005. 10.1016/j.transproceed.2004.12.137 15848514

[11] Carrero JJ, Hecking M, Chesnaye NC, Jager KJ (2018). Sex and gender disparities in the epidemiology and outcomes of chronic kidney disease. Nat Rev Nephrol.

[12] Lepeytre F, Dahhou M, Zhang X, Boucquemont J, Sapir-Pichhadze R, Cardinal H (2017). Association of sex with risk of kidney graft failure differs by age. J Am Soc Nephrol.

[13] Gupta A, Mahnken JD, Johnson DK, Thomas TS, Subramaniam D, Polshak T (2017). Prevalence and correlates of cognitive impairment in kidney transplant recipients. BMC Nephrol.

[14] Jay CL, Washburn K, Dean PG, Helmick RA, Pugh JA, Stegall MD (2017). Survival benefit in older patients associated with earlier transplant with high KDPI kidneys. Transplant..

[15] Haider M, Yessayan L, Venkat K, Goggins M, Patel A, Karthikeyan V, editors. Incidence of contrast-induced nephropathy in kidney transplant recipients. Elsevier. Transplant Proc. 2015;47(2):379–83. . 10.1016/j.transproceed.2015.01.008 25769577

[16] Goldberg I, Krause I (2016). The role of gender in chronic kidney disease. EMJ..

[17] Loor G, Brown R, Kelly RF, Rudser KD, Shumway SJ, Cich I (2017). Gender differences in long-term survival post-transplant: a single-institution analysis in the lung allocation score era. Clin Transpl.

[18] Schoening WN, Helbig M, Buescher N, Andreou A, Bahra M, Schmitz V (2016). Gender matches in liver transplant allocation: matched and mismatched male-female donor-recipient combinations; long-term follow-up of more than 2000 patients at a single center. Exp Clin Transplant.

[19] Yanishi M, Tsukaguchi H, Huan NT, Koito Y, Taniguchi H, Yoshida K (2017). Correlation of whole kidney hypertrophy with glomerular over-filtration in live, gender-mismatched renal transplant allografts. Nephrol..

[20] Puoti F, Ricci A, Nanni-Costa A, Ricciardi W, Malorni W, Ortona E (2016). Organ transplantation and gender differences: a paradigmatic example of intertwining between biological and sociocultural determinants. Biol Sex Differ.

[21] Pawarode A, Fine DM, Thuluvath PJ (2003). Independent risk factors and natural history of renal dysfunction in liver transplant recipients. Liver Transpl.

[22] Kovesdy CP, Trivedi BK, Anderson JE (2006). Association of kidney function with mortality in patients with chronic kidney disease not yet on dialysis: a historical prospective cohort study. Adv Chronic Kidney Dis.

[23] Meier-Kriesche H-U, Kaplan B (2002). Waiting time on dialysis as the strongest modifiable risk factor for renal transplant outcomes: a paired donor kidney Analysis1. Transplant..

[24] Ball AM, Gillen DL, Sherrard D, Weiss NS, Emerson SS, Seliger SL (2002). Risk of hip fracture among dialysis and renal transplant recipients. Jama..

