# Correction to: Impact of the donor-recipient gender matching on the graft survival from live donors

**DOI:** 10.1186/s12882-020-02148-2

**Published:** 2020-11-16

**Authors:** Gholamhossein Naderi, Amin Azadfar, Seyed Reza Yahyazadeh, Fatemeh Khatami, Seyed Mohammad Kazem Aghamir

**Affiliations:** 1 Shariati Hospital, Department of Urology, Tehran University of Medical Sciences, Tehran, Iran; 2 Urology Research Center, Tehran University of Medical Sciences, Tehran, Iran

**Correction to: BMC Nephrol (2020) 21: 5**

**https://doi.org/10.1186/s12882-019-1670-x**

Following publication of the original article [1], the authors would like to correct the affiliation number 2.

The incorrect affiliation currently reads:

^2^Urology research Center, Hassan Abad Sq., Sina Hospital, Tehran University of Medical Sciences, Tehran, Iran

The correct affiliation should read:

^2^Urology Research Center, Tehran University of Medical Sciences, Tehran, Iran

The original article has been corrected.
